# Critical issues for employees in inter-municipal health care services: a multiple case study

**DOI:** 10.1186/s12913-018-3586-8

**Published:** 2018-10-22

**Authors:** Elisabeth Holen-Rabbersvik, Tom Roar Eikebrokk, Rune Werner Fensli, Elin Thygesen, Åshild Slettebø

**Affiliations:** 1 Faculty of Health and Sport Sciences, University of Agder, PO Box 509, Jon Lilletunsvei 9, 4898 Grimstad, Norway; 2 Faculty of Social Sciences, University of Agder, PO Box 422, Universitetsveien 25, 4630 Kristiansand, Norway; 3 Faculty of Engineering and Science, University of Agder, PO Box 509, Jon Lilletunsvei 9, Grimstad, 4898 Norway; 4 Faculty of Health and Sport Sciences, University of Agder, PO box 422, Universitetsveien 25, 4630 Kristiansand, Norway

**Keywords:** Inter-municipal cooperation (IMC), Case, Cooperation, Municipality, Health, Reform, Employee

## Abstract

**Background:**

Traditional, hierarchical government structures have recently been challenged by increased complexity, fragmented services and heavy public demand. When healthcare services become fragmented and decentralised, they require redesign. Inter-municipal cooperation is a strategy to deal with current challenges and future demographic changes. Few studies exist that can help us conceptualize challenges regarding employment in this context and inform managers in the involved municipalities. This study aims to identify critical issues for employees in inter-municipal health care services and to elaborate on how and why these issues are experienced.

**Methods:**

A multiple qualitative case study was conducted with data from interviews, observation studies, a participant workshop and inter-municipal healthcare service project documents and reports. The study involved two districts in Norway and six cases including 17 informants. First, a within-case analysis was conducted for all cases; second, a cross-case analysis was conducted in each district to examine replication, contrasts and extension to emergent findings; and, eventually, replicated findings in Districts 1 and 2 were analysed across districts.

**Results:**

Three critical issues were identified: support, differences, and geographical distances. Employees working in teams experienced fewer challenges than did those working as isolated individuals.

**Conclusions:**

Critical issues for employees represent an important aspect of inter-municipal cooperation, and additional research should be undertaken to inform future policy and practice.

## Background

In recent decades, traditional, hierarchical government structures have been challenged by increased complexity, fragmented services, heavy public demands and the need to reduce costs [1, 2]. In health care services, factors like patient multi-morbidity, changing patient expectations, economic pressure, technological development and predicted decreases in the working-age population contribute to increasingly complex challenges for effective health care service production and delivery in local governments. Health care service delivery must be scaled to meet future needs, which will require changes that include service redesign and new structures for public service provision. Within local governments, inter-municipal cooperation (IMC) is one strategy used to meet future changes in the public sector [3, 4]. In contrast to a traditional, hierarchically organised municipality, IMC often represents a less formalised and more loosely coupled system where control is distributed across several municipalities. This might create challenges in coordinating management and control across municipalities in general and might create uncertainties for the employees in particular. Hence, IMC represents an additional dimension of complexity within municipal health care services. Previous research has noted the need for inter-organisational integration in public health services [5], and research focusing on public networks are increasing [6]. To shed a light on the complexity of working in an inter-municipal healthcare service, two theoretical lenses are of particular relevance; Governance networks and Perceived Organisational Support (POS).

### Governance network

IMC has been described as involving contracts or joint production with other local governments. The goal is to gain economics of scale, improve service quality, and promote regional service coordination across fragmented local government regions [7, 8]. IMC in various municipal service deliveries are a widespread phenomenon, particularly in small municipalities [9–11]. Inter-municipal cooperation can be seen as a so-called governance network. Governance networks can be defined as “more or less stable patterns of social relations between mutually dependent actors, which form around policy problems and/or clusters of means and which are formed, maintained and changed through a series of games” [12], and refers to a horizontal interaction between autonomous, yet interdependent actors [13].

In the last decades, network organising, in contrast to traditional hierarchical structures, has become more popular. Flexibility and the ability for rapid adaptation are found to be among the greatest advantages of networks [14]. It has also been expressed that it can change democratic processes found in traditional hierarchical organisations, both in a positive and a negative way. It can provide an alternative way for stakeholders to achieve democracy. Sørensen and Torfing [15] express that “…governance networks provide a supplement to representative democracy that grants an extra channel of influence to those who are intensely affected by certain decisions” [15]. In an inter-municipal health care setting, this will imply that patients and users of the services potentially will have more influence on the service. Moreover, it is expressed that contemporary problems in public sector requires flexibility, and that governance networks potentially has the advantage to be able to self-organize [16]. This implies more power, autonomy and influence for employees in IMC. Such organisational democracy can be both positive and negative for the employees. Potential advantages for employees are enhanced commitment, the feeling of more responsibility and a more participatory climate overall [17]. Potential disadvantages are the increased demands and accountability such a change of power can imply for the employees [17]. On the other hand, network governance has also been found to represent a threat to democracy, when democracy is defined from theories of liberal democracy such as Habermas [18] and Rawls [19] and described by Sørensen and Torfing [20]. Literature on IMC specifically, has identified challenges involving the potential democratic deficit of such cooperation [11]. Questions can be asked about whether local councils are informed about decision-making processes and budget control in inter-municipal service delivery [11]. However, if one sees democracy from the view of new post-liberal theory such as that of Etzioni [21] and Young [22], their emphasis is on the need for both a horizontal and vertical balance structure, and hence governance networks can play a positive role in democracy [20].

### Perceived Organisational support

The concept of POS can shed light on employees and their influence in IMC settings. In general, employees perceived organisational support (POS) is found to positively influence affective commitment, job satisfaction, positive mood at work, desire to remain with the organisation, and turnover intentions [23]. POS consist of employees’ common global perception concerning the extent to which the organisation values their contribution and cares about their well-being [23]. For the employees, the organisation gets humanlike characteristics that lead to the perception that the organisation has a favorable or unfavorable orientation against them [24]. Based on organisational support theory [24], the organisations’ favorable treatment regarding fairness, supervisor support and organisational reward and job conditions will most likely increase POS, and hence how employees perceive how their contribution is valued and their well-being cared for in the organisation [25]. POS has also been addressed regarding change related uncertainty in organisations [26]. POS was found to be a mediator of the relationship between employee’s adaptability and perception of change-related uncertainty and employees’ satisfaction and performance [26]. POS lead to responsible behavior such as knowledge sharing behavior that benefits the organisation [27]. Knowledge sharing is important for the fulfilment of common goals amongst cooperative partners [28]. Trust in supervisor is also found to be of importance for knowledge sharing behavior [27].

### Research questions

In inter-organisational theory, the focus is primarily on the collaboration across organisational boundaries, however, the employees are also part of the partnership and network they inhabit [29]. Despite an extensive focus on coordination and collaboration in health care services, as well as on inter-organisational coordination and IMC, few studies have focused exclusively on IMC in health care services, and there is a lack of contextual grounding on research and theory on employees in this setting. As a result, we do not know how to manage and coordinate the work of health care personnel in this setting in order to meet their needs involved in the production of current and future health care services. The role of employees in improving service production has for long been a topic in the management literature (e.g. C Grönroos and P Voima [30]) but few studies have addressed how and under what conditions employees can contribute to improved service production in general (e.g. J Gummerus [31]) and in particular to inter-organisational health care service production. To address this gap, this study aims to explore critical issues for employees in inter-municipal health care services. This will have important implications for personnel management in inter-organisational cooperation’s in public health care services. The aim is elaborated in the following research questions:

RQ1: What critical issues are experienced by employees working in inter-municipal health care services?

RQ2: How and why do inter-municipal employees experience the identified critical issues?

RQ3: How do identified critical issues affect employees and working practices in inter-municipal health care services?

Furthermore, similarities and conflicts in inter-disciplinary theory on network governance and POS are examined, and thereby contributing to managerial and theoretical enhancement.

## Methods

### Study design

An inductive multiple qualitative case study strategy was applied. Employees with different experiences of IMC were included to develop a rich dataset. According to Yin, “A case study is an empirical inquiry that investigates a contemporary phenomenon in depth and within its real-life context, especially when the boundaries between phenomenon and context may not be clearly evident” [32]. The focus of present study was the critical issues for employees in newly established inter-municipal health care services. Because of its strong relation with the context, a case study approach was established. Further, a multiple case study was established to get the analytic benefit of applying replication across the cases in various contexts, and hence increase analytic generalisability. Multiple case studies are more parsimonious than single case studies, but also more robust and generalizable [33]. Qualitative data from individual and focus group interviews, observational studies, workshops and several reports were collected, and a cross-case analytical method to yield contextually grounded and generalisable findings was adopted [34].

### Setting

The study took place in Norway, a country with three levels of political administration: state, county and municipal. In addition to these three formal administrative levels, Norway is divided into districts, which are organised by culture, common language or geography. The districts are larger than the municipalities but smaller than the counties and may span county borders. The districts in the present study are formal districts with a council and representatives from each participating municipality. Norway implemented the “Coordination Reform” in 2012 [35]. Because this reform entails the delivery of more health services via local governments, and because many municipalities are sparsely populated, the need for IMC has increased.

The participants in the interviews, observational studies and workshop came from two districts in Norway. The districts were chosen because both had newly established inter-municipal health services and hence were comparable in terms of the inter-municipal services. The contexts varied; one district comprised a mix of coastal and inland municipalities and the other comprised only inland municipalities. They differed in numbers of municipalities and population, enabling us to provide findings applicable across different contexts.

District 1 contained four municipalities with less than 10,000 inhabitants in total. The district council had established a health project grounded in the coordination reform [35]. The health project established several health-related inter-municipal projects, all rooted in the district council steering committee.

District 2 contained six municipalities with a total of 35,000–40,000 inhabitants. To adapt to the “Coordination Reform” [35], the district established a district health network that comprised managers of all six municipalities, the district hospital and the regional user committee, and union representatives. The main objective of establishing this network was to facilitate health care cooperation between the municipalities and between the municipalities and the hospital. One of the mandates of the network was to initiate and steer inter-municipal health care services.

### Participants

Six inter-municipal health services, representing the six cases were included in the study; inter-municipal dementia team, psychologist, substance abuse therapist, occupational therapist, palliation project and substance abuse team. The six cases involved 12 employees of the inter-municipal health care services and five collaborating partners such as community nurse, general practitioner and inter-municipal ICT-personnel. The research team contacted managers in the district, who identified participants meeting the following inclusion criteria: project managers and employees in an inter-municipal public health care service. In addition, employees working in health care services, managers, ICT-personnel and collaborative health personnel in the municipalities, were included. Altogether, seventeen persons participated in the study (Table 1), including six who participated in multiple aspects of the study, such as interviews, observational studies and workshop. This approach was adopted to ensure an in-depth understanding of the phenomenon. Interview and observational data were collected over eight months. Pseudonyms are used to denote districts, municipalities, teams and the individual informants to protect participant privacy.

**Table 1:** Participants

	Profession	Sex	Age	Data collection methods
District 1	Psychologist	Female	20–29	● Qualitative interview
	Substance abuse therapist		20–29	● Qualitative interview
	ICT manager	Female	40–49	● Qualitative interview
	Dementia coordinator	Male	50–59	● Workshop
				● Focus group interview
				● Two observational studies
	Dementia contact 1	Female	50–59	● Workshop
				● Focus group interview
	Dementia contact 2	Female	50–59	● Workshop
				● Focus group interview
				● Observational study
	Dementia contact 3	Female	20–29	● Workshop
				● Focus group interview
	Dementia contact 4	Female	30–39	● Workshop
				● Focus group interview,
				● Observational study
	Consulting doctor	Male	30–39	● Workshop
				● Focus group interview
	General practitioner	Female	30–39	● Qualitative interview
	Community nurse manager 1	Female	30–39	● Qualitative interview
	Community nurse manager 2	Female	20–29	● Qualitative interview
	Project manager for substance abuse therapist and psychologist	Female	50–59	● Qualitative interview
District 21.1.1.	Occupational therapist	Female	30–39	● Qualitative interview
	“Palliation in Vik” project manager/coordinator	Female	30–39	● Qualitative interview
	Manager of a substance abuse team	Female	30–39	● Qualitative interview
	ICT consultant	Female	30–39	● Qualitative interview

### Inter-municipal services

The inter-municipal health care services in this study were mainly organised as projects with fully or partially external public funding. The services were organised either on the basis of inter-municipal teams or as individual employees serving several municipalities (hereafter “individual service”). All the services were newly established from three months to 1.5 years prior to the start of the study.

In District 1, the dementia team was structured such that the employees and the project manager carried out their main work in their respective municipalities with part-time inter-municipal employment. The substance abuse therapist and psychologist had a 100% inter-municipal position each and served all four municipalities. One municipality served as the host municipality and fulfilled management responsibilities.

In District 2, the project “Palliation in Vik [anonymised district name]” was established as a competence-enhancing project for district health personnel financed by external public funding. Only the role of project leader had an inter-municipal component (30%) for the coordination of competence-raising measures. The occupational therapist was employed in a service that entailed her serving the involved municipalities individually. This position was administratively organised by one of the municipalities, whereas the hospital was responsible for professional supervision. The Vik substance abuse team consisted of four positions, including a project manager. One of the municipalities, the one in which the team was based, served as a host municipality.

### Data collection

Multiple data collection methods such as interviews, observational studies, workshop and document studies were used to strengthen the theoretical grounding by triangulating the evidence [36].

#### Interviews

A total of 11 individual interviews and 1 focus group interview were conducted in the two districts. The interviews were loosely structured based on a semi-structured interview guide dealing with inter-municipal employment. The main questions dealt with what and how their work was done and how the inter-municipal work tasks were performed, by whom and how participants collaborated in the work they performed, as well as how information was documented and shared. The municipal employees were asked about their work tasks and their relationships with the various inter-municipal services. All interviews were audio recorded and transcribed except for one interview where notes were taken. In District 1, seven individual semi-structured interviews lasting from 20 min to two hours were conducted. One focus group interview was also conducted in District 1. Five participants participated in the focus group interview, which was divided into two sections of one and one-half hour each. In District 2, four individual semi-structured interviews, lasting from 30 min to 45 min were conducted.

#### Observational studies and workshop

Two observational studies were conducted, following the dementia team’s work; from the referral of a patient, the preparation in the office, the dementia assessment in patient homes, the patient documentation and the referral to collaborative partners. The observations were conducted as open observation in all cases, aimed at understanding the context of the employees working in IMC. The observation studies were conducted in everyday settings including informants’ offices, patients’ homes and transport between office and patient homes. Observations in the patients’ home were conducted as nonparticipative observation to not interrupt the patients’ assessment. The researcher was sat at a distance from the patient and health personnel and did not interrupt or ask questions. Questions regarding how and why tasks were done, were asked in front of and after the assessment. In the other settings, the observations were conducted as participant observation, and questions were asked regarding how and why participants performed tasks. The questions were asked to ensure a complete understanding regarding how and why tasks were done. The combination of nonparticipative and participant observations provided *an in-depth understanding of the employees work and the context of the service and permitted comparison between the interview data and observations. In District 1, a* workshop aiming at validating and get increased insight on work procedures were conducted. Eight participants were present; seven participants from an inter-municipal team and one cooperating partner. The workshop was audio recorded.

#### Documents

To corroborate the empirical data, seven local project/evaluation reports and plans related to the different services were collected; three from District 1 and four from District 2. In District 1, the inter-municipal ICT service was organised as a project; and a pilot study and project report were included in our data.

### Data analysis

**Fig. 1:**
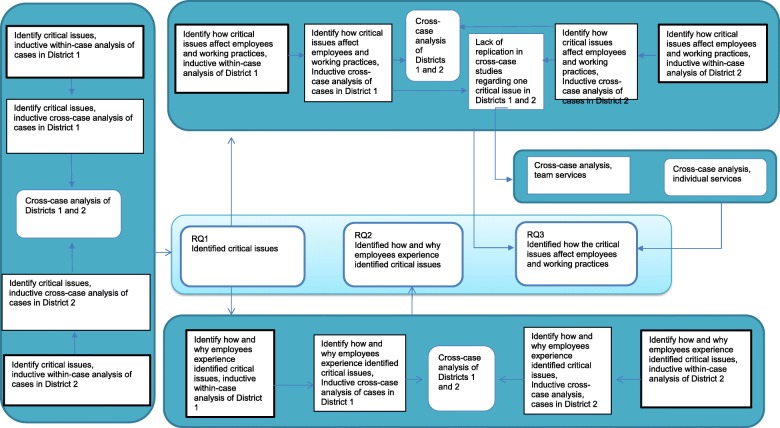
“Data analysis process”

The data analysis was conducted in iterative phases, and the research questions and findings obtained during the process revealed a need for different approaches as visualized in Fig. 1.

The qualitative software program Nvivo [37] was used for the analysis. The research questions guided the analysis:

RQ1: What critical issues are experienced by employees working in inter-municipal health care services?

First, case analysis was conducted on all individual cases. Data sources relevant to each case were analysed using qualitative content analysis, as inspired by Graneheim and Lundman [38] and recommended by Kohlbacher [39]. The content was analysed through a series of readings of data relevant to each case. The data were analysed and developed inductive codes and categories based on the research questions. Examples are seen in Table 2. Second, cross-case analysis was conducted on the cases inter-municipal dementia team, psychologist and substance abuse therapist in District 1 and on the cases occupational therapist, palliation project and substance abuse team in district 2. The cross-case analysis in each district examined replications of, contrasts with, and extensions to emergent findings. Third, findings replicated across cases in both districts were analysed across the two districts to identify replications of, contrasts with and extensions to emergent findings. The initial findings of the present study are the categories replicated across cases within a district, and across districts.

**Table 2:** Illustration of data coding

Quotation	Code	Category
“...To have some arenas for cooperation, where you meet and can discuss those things, evaluate along the way, I think that is really important to in a way see if this is working, shall the tasks be changed, I came close to saying, is this what I’m supposed to be doing?”	Need for supervision	Support
“…But I do feel that it would have been nice if it could all be organised pretty much the same way. It is a lot to deal with when there are four different interfaces in the system [Electronic Health Record], different templates and different…”	Differences between municipalities	Differences
“…But the time, driving that many hours a week, I think it is a challenge. You lose a lot of time, it might be two hours of your workday and you don’t get paid for it…”	Additional practical challenges	Geographical distance

The categories served as a framework for addressing the following research question:

RQ2: How and why do inter-municipal employees experience the identified critical issues?

The same strategy, qualitative content analysis, was used in the further analysis of the data. Each category was analysed individually within each case using an inductive approach. Cross-case analysis was conducted within each district. Several local conditions exist regarding each identified category from RQ1, and replication logic across all cases was not obtained. However, the most important and prominent categories were identified. Finally, findings from each district were analysed, and a common theme was identified and considered as evidence for accomplishing literal replication.

RQ3: How do identified critical issues affect employees and working practices in inter-municipal health care services?

Each category from RQ1 was analysed individually within each case, using an inductive approach. Cross-case analysis was conducted within each district. The cross-case analysis revealed few to zero replications across cases in the various regions regarding the identified critical issue "support", which compelled the researchers to refine and extend the analysis approach. The findings indicated differences in how both teams and individual services were affected. Hence, the cases composed as teams and those composed as single services were analysed across cases but not across districts. The cross-case analysis in each group obtained findings that were replicated across cases. The replication logic confirmed the refinement of our analysis and suggested that service composition had more impact than district characteristics on how critical issues affect employees in inter-municipal services. Regarding the identified critical issues "differences" and "geographical distance", common themes were identified and considered as evidence for accomplishing literal replication. 

### Data validity and reliability

To obtain robust findings, a multiple-case design that allowed replication of findings was conducted. Data analysis revealed that no further replication was feasible, contributing to data saturation. The discussion actively uses existing literature from other contexts or viewpoints to enhance internal validity and analytic generalisability.

Both participant and nonparticipant observation can influence the participants’ behaviour. To reduce this potential bias, multiple data collection methods are used in the analysis of data.

The nature of case study design enables the transfer of findings to other contexts. The findings in the present study were replicated across two districts with diverse characteristics, indicating applicability to other settings and groups.

The research team consisted of multiple investigators, with multi-disciplinary competence. The complementary insights provided by a team can improve data richness and increase the likelihood of capitalising on novel insights from the data, while convergence from multiple investigators improves confidence in the findings [36].The prior knowledge of the researchers might affect the research process. The researchers avoided thinking about specific relationships between theory and findings during the data gathering and analysis. The participants varied in terms of age and gender, thus reflecting diverse perspectives on IMC. The constant use of different perspectives from researchers, various data collection methods and cross-case analysis comprised the strategy to avoid a unilateral focus, and hence researcher bias.

The data collection evolved over time, and the initial interviews gave new insights that influenced the decision to broaden the research question and made it necessary to include more participants to ensure saturation, and, hence, replication between multiple perspectives. The research team had open discussions regarding data consistency. To reduce the threat of selective bias, external colleagues working in inter-municipal health care services were consulted to check the possibility of rival or other reasonable explanations. In case studies, the extensive use of empirical evidence can result in too much reliance on theory with excessively detailed findings, and a lack of overall perspective [36]. Rather than trying to capture everything, this study focuses on findings that were replicated across multiple cases and in different contexts and avoids explaining rival findings across cases.

## Results

The overall finding was that support, differences in working practices between the involved municipalities and considerable geographical distance are critical issues for inter-municipal employees. Organisation of IMC in a team or at the level of individual services was found to influence how the identified critical issues affect employees. Team members appear to use each other’s resources for supervision and support and are capable of taking advantage of autonomy related to the identified issues. Hence, IMC organised at the level of individual employees involves more challenges than does IMC organised at the team level. In the following sections, the three critical issues will be elaborated on through quotes and explanations and divided according to the research questions asked.

### Support

#### Critical issues

The findings suggest that support is a critical issue for inter-municipal employees. Both supervisor support, here defined as support from an employee’s immediate superior, and co-worker support were found to be important. Team members largely took advantage of co-worker support within the team. In addition, the findings made it clear that inter-municipal employees themselves acted as experts, with professional supervision of municipal employees being one important task. However, the inter-municipal employees were found to lack supervisor support in relation to both professional and organisational issues. The lack of support was related to professional feedback regarding direct patient cases and clinical themes, and administrative support regarding the execution of work.

The existence of several different managers having different local needs poses a challenge for the inter-municipal employee. The need for regulated arenas to ensure evaluation, and to ensure the employee is focused on the right tasks, is elaborated as follows by the inter-municipal employee:

...to have some arenas for cooperation, where you meet and can discuss those things, evaluate along the way, I think that is really important to, in a way, (to) see if this is working; shall the tasks be changed? I almost said, is this what I’m supposed to be doing?

Services provided through IMC were specialized municipal services. Therefore, relatively few municipal employees were trained in delivering these services, leading to only limited discussions regarding service-related matters. In some cases, there was communication with specialist health care services to provide necessary supervision and professional support, and it was decided that this communication had to be on a regular basis.

In addition to the lack of professional discussion partners for individual inter-municipal employees, the lack of supervisor support regarding the actual execution of assignments was a challenge. This resulted in uncertainty regarding whether the work was done as expected by the representative of the employing municipalities.

For the team members, the lack of supervisor support was not explicitly conveyed. During data collection, it became apparent that team members managed their work by providing professional supervision to one another. Decision-making processes were influenced by collaboration among horizontal partners in the municipalities and end-users. In none of the cases were upper management or immediate superiors highlighted in the descriptions of how team members performed their work or made decisions on what to do and how. One example is that the team members used each other as discussion partners and supervisors on patient cases. One team member in team A stated:

But I do feel that I get a lot of competence from the other team members, just by discussing different cases, challenges, things I find difficult. So, I get a lot of suggestions, and yes, at least that is competence for me.

Team B worked as its own supervising mechanism. They had regular weekly meetings where challenging cases were discussed. This served as an arena for supervision.

Decisions on how to perform tasks, as well as what tasks to perform, were guided by the needs of end-users and collaborating partners in a bottom-up strategy. Team A established a “relatives café” based on needs conveyed by relatives.

#### How and why employees experience support as a critical issue

The findings indicate that inter-municipal employees in health care services lack overall governance. This situation relates to the lack of support experienced by these employees. Britt mentioned that in one municipality, the health care manager did not display a conscientious attitude regarding his role in IMC:

…the person to whom, in a way, the health care manager that I am supposed to belong to, when I asked him “are you my immediate professional manager?” Well, no he was not sure about that. So, I do not know, well, it is very unclear.

The findings indicate that roles are not properly clarified prior to their establishment and that the service is not strongly anchored in the municipalities. This lack of support creates a challenge for the employees, as they do not know who they shall contact regarding services they are delivering in the municipalities.

#### Effect on employees and working practices

The findings revealed a distinct difference between how a lack of support influenced inter-municipal employees organised in teams as opposed to those organised in individual services. Team members effectively used each other for supervision, and adapted services based on feedback from collaborators and service users.

#### Teams

The lack of support in the teams was not emphasized, and the findings suggest team organisation was safeguarded by co-worker support and feedback and user feedback rather than by external support. Findings suggest that the teams took advantage of their autonomy and used a bottom-up strategy to adapt services based on the interests of partners and users, and to improve their own working conditions. Distance from supervisors created the opportunity to deviate from the regular bureaucratic decision-making. For services offered via an organised team structure, this lead to flexibility and adaption based on interaction among municipalities, collaborators and user needs. Such flexibility benefited service users and was valued by municipalities involved in IMC. In a project report it is stated: “The municipalities experience it [the service] as useful, both in individual follow-up work, where the team has shown to be very flexible, and they see it as a resource concerning general advisory in the field [of expertice].”

Municipalities that need inter-municipal cooperation in health services are often sparsely populated, meaning there is a high likelihood of personal relationships existing between employees and users. Sometimes this creates challenges owing to problems in these personal relationships. In such cases, the inter-municipal teams use their flexibility and seek to match users with the most suitable employees.

Employees in teams emphasized the feedback of users and relatives and aimed to meet their needs and wishes in a quick and adaptive manner. A team member states: “…The users provide the feedback that when things happens through the dementia-team, they happen very quickly…”.

#### Individual services

The individual employees were afflicted by not having support in terms of both professional and organisational feedback. They felt isolated in their work and stated that it was difficult to achieve satisfactory cooperation; as Britt stated: “I don’t know, but I can easily be alone at work, so that is a challenge. So I think it requires a lot from me, to manage to cooperate.” Without any possibility of a professional discussion partner, their work gave them little opportunity for development. Gunn stated: “… because I find the position very lonely, and I get little stimulus in a way, and I need that. Somebody to discuss difficult cases with”.

Expressions conveying autonomy in their work were not present in data from employees in individual services. Unlike the teams, they were supposed to deliver the service on their own and experienced little room for flexibility in terms of service organisation; hence, autonomy was not utilized.

### Differences

#### Critical issues

Employees experienced differences among collaborating municipalities concerning management and organisation of health care services, as well as in terms of competence and resources such as the electronic health record (EHR). There was no consistency among the municipalities in terms of service organisation, leading to different procedures in work routines, documentation practices and contact persons. In a joint-service provision like IMC, employees had to use solutions specific to the various municipalities.

The employees emphasized the differences in EHR management. Some municipalities obtained their EHRs from various vendors, while others shared a common vendor but still their EHRs presented different information. Different organising within municipalities also created challenges related to the physical location of inter-municipal employees because secured health networks were not available in the welfare units. The differences also applied to resources and competence among the municipalities involved in the cooperation.

#### How and why employees experience differences as a critical issue

Because the municipalities differ and have individually adapted services, it is challenging to devise effective solutions that fits all. In both cases, substance abuse work was organised in health care units in some municipalities, and in welfare units in others. In District 2, the municipalities had chosen various EHR vendors. In District 1, despite a conscious decision to implement an EHR system from the same vendor, the system content nevertheless varied among the municipalities. The Norwegian language consists of two written language variants, and in District 1, the written language used was different depending on the cooperating municipalities, and this led to further challenges.

Ellie emphasized the importance of employees not being constrained by the municipality in which a patient lives. However, the jurisdiction and guidelines were contradictory. Lack of national guidelines made it difficult to devise good solutions for all municipalities. Differences between municipalities also related to the need for new systems to conduct the inter-municipal services. The overarching solutions necessary to support inter-municipal employees were often not in place before service establishment.

The municipalities varied in how geographically central they were, and in an evaluation report for the dementia team it was stated that geographical location could affect access to relevant competence: “both to recruit and keep qualified personnel is an extra challenge for the inland municipalities.” In Norway, the larger municipalities are mainly coastal towns. Inland municipalities are hence more rural, and this affects access to competence and skills.

#### Effect on employees and working practices

The differences caused increased system complexity and workload and decreased efficiency and effectiveness. Employees expressed a wish for a more uniform way of organising the services because the differences created additional work burden. The differences were also related to how implementation of necessary ICT solutions was prioritized in the municipalities. This prevented employees from documenting health services provided and caused a lot of frustration.

The differences between competence and resources in the municipalities resulted in two opposite consequences for employees. In one case, the differences resulted in additional work for the employee: “I have been dragging for Berg [a Municipality] for several years, because they have not had the competence, and that is okay, but we have had to share and share, and share…”.

In one of the districts, the differences in competence between municipalities resulted in the investment of unequal effort in IMC. However, in the other district, the differences in competence between municipalities resulted in less use of inter-municipal employees. The two municipalities that lacked occupational therapy services were found to be the ones not using the service.

### Geographical distance

#### Critical issues

The findings suggest that geographical distance was a critical issue for employees, albeit mainly a negative one. A large proportion of part-time, inter-municipal jobs involve travelling long distances. When discussing the time spent on a patient assessment, a project leader mentioned that a single assessment could involve hours spent driving. If the assessment were carried out in a municipality far from the employee’s main working place, it could take an entire day. As the project leader put it: “… and some hours driving too maybe, so if I’m going to municipality C, it actually takes a day.”

#### How and why employees experience geographical distance as a critical issue

Some inter-municipal employees spend time working in municipalities other than those where their offices are located. This leads to personal challenges in terms of their work attendance in different municipalities. Different municipalities can be separated by long distances, and employees must sometimes spend a great deal of time simply getting to their work place. Natalie states: “…But the time, …driving that many hours a week, I think it is a challenge. You waste a lot of time; it might be two hours of your workday, and you don’t get paid for it…”.

The findings also indicate that roadway infrastructure relates to the challenges of distance, with poor infrastructure increasing the challenges of long distance. However, with effort, the challenges experienced by employees could be decreased. A project report for the substance abuse and psychologist service states that the employment situation should be improved, implicitly indicating that managers can implement various measures to influence how driving distances affect employees.

#### Effect on employees and working practices

On a personal level, the distance was challenging because potential spare time was used to travel to work; meanwhile, on a professional level, there were challenges in that working time was used for travel rather than actual work. Some inter-municipal services were supported only on a part-time basis. Employees spending only 10% of their working time on IMC expressed that much of this limited working time was consumed by long distances: Erna states “Long distances […] consuming a lot of the ten percent [part-time job] we got”. This suggests that employees have to spend a disproportionate amount of their working time on driving. In an annual report in District 1, driving distance was considered the most challenging aspect of the service for two of the inter-municipal employees, and the main reason for one of them quitting the job.

Geographical distance also affects how employees collaborate and with whom one collaborates. Britt stated:

...Here it is easier to drop by, and the same is true in municipality C; he is the one I have collaborated with the most, and he is located at the neighbouring office, so it’s just next door, so that is of relevance, how close they are and how you are able to collaborate.

The findings suggest that distance is important, and proximity stimulates informal contact.

## Discussion

This study aimed to identify the critical issues for employees when working in an inter-municipal health care service, and to elaborate how and why these issues were identified and how they affected employees. The findings revealed three critical issues: support, differences and geographical distance. Several findings indicated that the use of teams in the inter-municipal organisation of service provision positively affected employees compared with situation in which individuals worked independently.

Lack of support was found to be a characteristic of inter-municipal services. Previous research suggests that direct management, which is based on continuous information flow between practitioners and their governing body, as in a traditional, hierarchical organisational structure, is lacking in horizontal cooperation like IMC [40]. The complex leadership in inter-municipal cooperation in health care services are found to be challenging [41]. According to the theory of perceived organisational support (POS), supervisor support is an important antecedent to POS [25], and hence impacts employee commitment, loyalty and performance [23]. Previous research emphasizes the negative effects of not receiving support from one’s immediate superior, and this is considered to be a predominant issue for horizontal networks like IMC. Lack of supervisor support implies lack of trust in supervisor. We do know that trust in supervisor promotes knowledge sharing behaviour in the organisation, which is an important goal for inter-organisational cooperation’s. Hence, the lack of support can potentially affect the cooperation negatively. However, the findings do suggest that the lack of support was not perceived as challenging for the team members. They emphasized the importance of support from other team members. Previous studies have suggested that teamwork reduces both the need for supervisor support in an organisation, as well as the importance of such supervisory support for individuals [42]. This suggests that although supervisor support is lacking in inter-municipal services, this might not be experienced as a hardship for team members.

Inter-municipal employees are often employed to manage specialized health care issues, in cases where a larger population base is necessary to build proper competence. Hence, employees in these positions are supposed to be experts on a specific field, serving in a municipality that lacks this competence. Inter-municipal employees thus act as supervisors rather than as supervisees. Supervisor support is more closely related to POS than co-worker support, because supervisors are seen as more representative of the organisation. Supervisor support implies an inter-level relationship, whereas co-worker support implies an intra-level relationship. Clinical supervision can be inter-level but is not exclusively so. Co-workers might also serve as supervisors. A definition of clinical supervision is as follows:

Clinical supervision is a support mechanism for practising professionals within which they can share clinical, organisational, developmental and emotional experiences with another professional in a secure, confidential environment in order to enhance knowledge and skills. GM Lyth [43].

In health care services, clinical supervision can be given equally from a co-worker as from a supervisor, explaining findings that team members did not need supervisor support from a manager. In contrast, employees in individual services experienced negative consequences from the lack of supervisor support.

The findings suggest that lack of supervisor support provides overall autonomy regarding what tasks should be performed and how, as well as the allocation of work within the team. This can relate to the implementation of new services in municipalities, and to the need for flexibility regarding the services that should be included in the implementation phase and how they should be included. However, the findings suggest that IMC organised in teams are most likely to take advantage of the flexibility given to them. Flexibility and the ability for rapid adaptation are found to be among the greatest advantages of networks [14]. In previous studies, autonomy in teamwork has been highlighted as important [44, 45] Employees in individual services have reduced possibility to be flexible or adapt the services provided because they lack others with whom to collaborate. Challenging governance and democratic control can be found in inter-municipal cooperation [15, 41]. However, how this affects employees, and hence service delivery, has not been determined. Employees mention the importance of feedback from municipal partners such as the General Practitioner (GP), as well as from patients and their relatives. For team members, this leads to a grounded fit of services, where end-user needs are guiding service content and structure.

Previous literature states that in a network, stakeholders can achieve democracy [15, 46]. The empirical findings support this theory and indicate that IMC can potentially foster a new dimension in representative political democracy, because employees are guided and take decisions based on a bottom-up strategy involving end-users. Network governance have been found to represent a threat to democracy, [18–20], but from the view of post liberal theories, findings in this study can be seen as an empirical evidence for the democratic potential in networks, despite the lack of governance [20–22]. Co-creation and co-production of public services is a response to the democratic deficit experienced the planning and deliverance of public services [47]. The users contribution is crucial to the performance of the service and creates value for the service user [47]. The inter-municipal teams develop services in cooperation with users, relatives and cooperative partners and such an organising of the inter-municipal service is a promising action to facilitate co-creation between employees and their stakeholders in public services.

The findings suggest that the municipalities differ in terms of economy, population density, EHR and several locally adapted solutions exist. This becomes a challenge when employees work across municipal borders. Cultural diversity is found to be challenging and can negatively impact identification of shared interests and understanding between cooperating partners [48]. While some studies treat boundaries as a means for ordering differences, other studies have conceptualized them as interfaces that facilitate knowledge production by enabling communication across organisations [49, 50]. However, clarity is known to be one important antecedent for job satisfaction [51]. The differences were negatively affecting the employees and led to additional work. Findings suggest that common procedures and tools are desirable to ensure clarity, and hence, job satisfaction in inter-municipal health services.

Geographical distance was also found to be a critical issue for employees. Driving long distances was challenging, yet inter-municipal services are often necessary in sparsely populated areas. Hence, inter-municipal services must organise service delivery over a relatively large geographical area. The vast majority of modern workers commute, and the number of long distance commuters seems to have increased [52]. For health care workers, the location of their workplace is dependent on where patients live, as well as political considerations. The relationship between long commuting distance, and the likelihood of quitting a job is well known [53]. An antecedent for job satisfaction are the work and family balance [51]. Findings indicate that the driving distance is challenging because free time is used to drive long distances. Findings suggest that managers recognize they can do something about this situation. Location-specific wage subsidies have been identified as one means to support commuting [52].

### Limitations

The sample size in the present study was small and the geographical setting was limited. This study does not emphasize or distinguish between different organisations and formalizations of IMC which can potentially affect critical issues related to IMC. Further research is needed to address these limitations

### Managerial implications

The findings of present study have some implications, which can guide managers of IMC in a health care setting. Employees were found to be affected by several critical issues, in which potentially can have negative impact on both job satisfaction and POS. This in turn, may affect turnover intentions and the employees’ perception on how the organisation values their contribution and cares about their well-being [25, 54]. In network governance theory, networks are described to be flexible, and they can change the existing democratic system [14, 20]. Findings in present study provides a contextually grounded support for these theories. However, the flexibility was only present in the services organised in team-structures, suggesting that the positive effect work across organisational borders are also dependent on how the employees are organised at the local level. Organising the employees in a team structure were found to hinder the negative impact of employment in inter-municipal health care services, and it could in addition better utilize the positive effects of IMC. Teams were more flexible in terms of their ability to match employees with suitable patient cases, as well as coping with time-schedules and distances. Team members made use of co-worker support and did not express challenges regarding lack of supervisor support. Future health care will be characterized by rapid changes and development. Thus, the ability to quickly adapt to environmental changes could potentially meet future needs. It was also apparent that employees in the inter-municipal services were guided and made decisions, based on a bottom-up strategy, supporting post-liberal theories suggesting that networks can enhance democracy by providing an additional channel for the stakeholder voice [21, 22]. Further, managers should be aware of the challenges related to differences amongst the cooperating municipalities, and the geographical distance. This lead to increased workload for the employees and decreased efficiency and effectiveness in the services. Managers should consider to putting in place measures to minimize the possibility of negative effects for the employees regarding geographical distance and differences amongst the municipalities.

The mandate of an inter-municipal service should be precisely explained, and flexibility within a defined framework can generate effective services with user involvement based on a bottom-up strategy.

## Conclusion

Critical issues for employees in inter-municipal services are found to be support, differences and geographical distances. Challenges related to lack of governance in inter-municipal services can explain why these work conditions were identified. This study is a contribution to the gap in the literature whereby focus has been exclusively on employees in IMC in health care services; the study thereby clarifies the need for an increased focus on critical issues in IMC in health care services from the perspective of the employees. In a public sector with an increasing amount of services being delivered across organisational borders, future research should elaborate on POS theory and focus on how employees can perceive organisational support in a context with many, and diverse, organisations. To elaborate on existing literature, future research should focus exclusively on employees in inter-organisational services. Findings indicate that potential negative effects from working across municipal borders, can be reduced by organising the service in a team structure. The employees in the inter-municipal teams can also facilitate co-creation of the planning and implementation of public services, and findings contribute to the emerging literature on co-creation value in public services. Therefore, future research should include both an organisational and an individual perspective when studying employment in inter-municipal health care services. In addition, this multiple level focus should be encompassed by a multi-disciplinary research approach to capture the complexity of the field. This study was a cross-case study across different districts within the same country. Finally, future research should focus on cross national study, and identify replication and contradictions across national borders to further develop the research area.

## Abbreviations

EHR: electronic health record
GP: general practitioner
ICT: information and communication technology
IMC: inter-municipal cooperation
POS: perceived organisational support
RQ: research question
